# Structural changes in the gut virome of patients with atherosclerotic cardiovascular disease

**DOI:** 10.1128/spectrum.01050-23

**Published:** 2023-12-05

**Authors:** Youshan Li, Jie Ma, Jinxin Meng, Shenghui Li, Yan Zhang, Wei You, Xulin Sai, Jianfeng Yang, Shuo Zhang, Wen Sun

**Affiliations:** 1 Department of Peripheral Vascular Diseases II, Dongzhimen Hospital, Beijing University of Chinese Medicine, Beijing, China; 2 School of Traditional Chinese Medicine, Beijing University of Chinese Medicine, Beijing, China; 3 Puensum Genetech Institute, Wuhan, China; 4 Department of Traditional Chinese Medicine, Beijing Friendship Hospital, Capital Medical University, Beijing, China; 5 Department of Acupuncture and Moxibustion, Beijing Hospital of Traditional Chinese Medicine, Capital Medical University, Beijing Key Laboratory of Acupuncture Neuromodulation, Beijing, China; 6 Key Laboratory of Health Cultivation of the Ministry of Education, Beijing University of Chinese Medicine, Beijing, China; 7 Beijing Key Laboratory of Health Cultivation, Beijing University of Chinese Medicine, Beijing, China; University of California San Diego, La Jolla, California, USA

**Keywords:** gut virome, atherosclerotic cardiovascular disease, viral function, viral auxiliary metabolic genes, fecal metagenome sequencing, gut bacteriome

## Abstract

The gut microbiota is an important risk factor and therapeutic target in atherosclerotic cardiovascular disease (ACVD), a leading cause of morbidity and mortality worldwide. However, alterations in the gut viral community and its contribution to ACVD have rarely been investigated. In this study, we characterized and compared the gut viromes from the fecal metagenomes of 214 patients with ACVD and 171 healthy individuals using a reference-dependent virome approach. We revealed that ACVD patients exhibited a significant increase in viral richness at the family level and a visible alteration in overall virome structure regardless of host sex, age, or body mass index. At the viral operational taxonomic unit (vOTU) level, we identified 105 vOTUs that significantly increased in abundance in ACVD patients and 60 vOTUs that increased in abundance in healthy controls. A majority (43.8%) of the ACVD-enriched vOTUs were predicted to infect *Streptococcaceae*, *Lachnospiraceae*, *Ruminococcaceae*, and *Enterobacteriaceae*, and *Streptococcaceae* had tight correlations with the corresponding gut bacterial species, whereas a considerable proportion (35.0%) of the control-enriched vOTUs were *Bacteroidaceae*, *Burkholderiaceae*, and *Lachnospiraceae* phages. Functional analyses revealed five viral auxiliary metabolic genes that differed in frequency between ACVD-enriched and control-enriched vOTUs. Moreover, we identified gut viral signatures for ACVD discrimination and achieved an optimal area under the receiver operator characteristic curve of 0.878 for distinguishing patients from healthy controls. Our results provide a comprehensive view of the ACVD gut virome, which may contribute to the development of novel diagnostic and therapeutic strategies for ACVD and additional relevant cardiovascular diseases.

**IMPORTANCE:**

Existing studies have found that there is a close relationship between human virome and numerous diseases, and diseases may affect the diversity and composition of the virome; at the same time, changes in the virome will in turn affect the onset and progression of the disease. However, the composition and functional capabilities of the gut virome associated with atherosclerotic cardiovascular disease (ACVD) have not been systematically investigated. To our knowledge, this is the first study investigating the gut virome in patients with ACVD. We characterized the structural changes in the gut virome of ACVD patients, which may facilitate additional mechanistic, diagnostic, and interventional studies of ACVD and related diseases.

## INTRODUCTION

Cardiovascular diseases (CVDs), a class of diseases that affect the heart and blood vessels, are a serious threat to the health of humans, especially middle-aged and elderly people. CVDs typically include hypertension, coronary artery disease, and heart failure; they are characterized by a high lifetime morbidity of more than 50% for both men and women (1, 2). As one of the leading causes of mortality, CVDs kill up to 17 million people worldwide per year (3). Accumulating evidence has demonstrated that the gut microflora plays an essential role in the etiology and progression of CVDs (4, 5). Changes in the gut microbiota were observed in recent studies in hypertension (6), atherosclerotic cardiovascular disease (ACVD) (7), atrial fibrillation (8), and chronic heart failure (9). These studies revealed that patients with diverse CVDs generally harbor a dysbiotic gut microbiota that is characterized by the overgrowth of certain pathogenic bacteria (e.g., Enterobacteriaceae and *Streptococcus* spp.) and the depletion of some beneficial taxa. Regarding the mechanism, some bacteria in the human gut can metabolize dietary choline and L-carnitine to produce trimethylamine (TMA) and further TMA N-oxide (TMAO), which promote atherosclerosis and cardiovascular disorders (10, 11). Moreover, modulation of the gut microbiota has shown prominent therapeutic effects to prevent CVDs (12, 13).

Although recent studies have focused on gut bacteria as the main components that impact host health, the gut viral community (or “gut virome”) has also shown profound effects on patients with obesity, type 2 diabetes (14), autoimmune disease (15 – 18), and gastrointestinal disorders such as inflammatory bowel disease (19) and colorectal cancer (20, 21). Han et al. showed that the gut viral composition was changed along with disease severity in hypertension patients, and they revealed that the hypertension-associated viral signatures had a discrimination power superior to that of bacterial signatures for identifying the patients and healthy controls (22). Consistently, Kim et al. showed that the gut virome was a potential regulator of the gut ecosystem in patients with pulmonary arterial hypertension (23). In addition, alterations in the gut viral profile in patients with other CVDs, including coronary heart disease (24) and stroke (25), have been preliminarily investigated with relatively small sample sizes. These studies implied a potential role of the virome in CVD, highlighting the need to systematically examine the composition and functional capacity of the gut virome in relation to this significant disease.

In this study, we performed a metagenomic-based characterization of the gut viral community in patients with ACVD. The fecal metagenome data set was downloaded from a previous study (7) on a total of 214 ACVD patients and 171 healthy subjects. The gut virome was profiled from fecal metagenomes and compared between patients and healthy controls, which revealed numerous viral compositional and functional signatures associated with ACVD. Moreover, the ability of viral signatures to classify ACVD patients and healthy controls was also explored.

## MATERIALS AND METHODS

### Subjects and data set

The fecal metagenomic data set of 385 samples from 214 ACVD patients and 171 healthy volunteers was downloaded from the European Bioinformatics Institute database under the accession code ERP023788. The ACVD patients had a remarkably lower proportion of females (25.2%) than the healthy controls (59.4%; Table S1). However, there was no significant difference in age (61 ± 10 vs 60 ± 10 years for patients vs healthy controls, Student’s *t* test, *P* = 0.548) or body mass index (BMI) (24.6 ± 3.5 vs 24.5 ± 6.8, Student’s *t* test, *P* = 0.842) between the two groups.

Raw metagenomic reads were qualified via fastp (26) with the options “-u 30 -q 20 -l 60 -y -trim_poly_g,” and human reads were then removed by matching the high-quality reads against the human genome GRCh38 (GCA_000001405.40) with Bowtie 2 (27), and details were provided in Table S1.

### Gut virome profiling and analyses

A gut virus catalog comprising over 67,000 nonredundant viral operational taxonomic units (vOTUs) was constructed from over 10,000 publicly available fecal metagenomes from the Chinese population (28). Protein-coding genes of the vOTUs were predicted using Prodigal (29) with the option “-p meta.” Taxonomic classification of vOTUs was performed based on protein sequence alignment to the combined database derived from the National Center for Biotechnology Information RefSeq database, the Virus‒Host database (30), the *crAss-like* protein sequences from Guerin’s study (31), and the viral protein sequences from Benler’s study (32). vOTUs were aligned against the combined database using DIAMOND (33) with the options “-query-cover 50 -subject-cover 50 -id 30 -min-score 50 -max-target-seqs 10.” A viral sequence was annotated to a viral family when over one-quarter of its proteins were matched to the same family. To search for the potential prokaryotic hosts of the viruses, the CRISPR spacers in the genomic sequences of the Unified Human Gastrointestinal Genome (UHGG) database (34) were predicted using MinCED (parameter “-minNR 2”) (35), and then the spacers were compared with blast with the vOTU sequences (“blastn-short” mode and bitscore > 50) to identify the phage‒bacterial host pairs.

To profile the gut viral community in the fecal metagenomes of ACVD patients and healthy controls, we mapped the high-quality reads of all samples into the UHGG database using Bowtie 2 (27) first. This was done in order to reduce bacterial sequences that could potentially interfere with virome analysis. The remaining sequences were then aligned into the gut virus catalog using Bowtie 2 with a nucleotide similarity threshold of 95% [a phylogenetic threshold for viral “species-level” definition (36)]. All sequence alignment outputs were merged to calculate the coverage of vOTUs in these populations using the coverage workflow in the SAMtools program (37). Only vOTUs with a coverage of >75% were selected for further analysis. Next, the abundance profile of vOTUs in each fecal sample was generated by aggregating the number of reads mapped to each vOTU, and only vOTUs with a coverage of >1% were considered valid and included in the sequential analysis. The feature table of read count was rarified using a lower quartile of sequencing depth across all samples, utilizing the *vegan* package in the R platform. The read count of the vOTUs was normalized to TPM (Transcripts Per Kilobase of exon model per Million mapped reads) by standardizing it. This involved dividing the read count by the length of the vOTUs and computing its relative abundance in each sample. The relative abundance profile at the viral family level was generated by aggregating the relative abundances of vOTUs assigned to the same family.

For each sample, the observed number of vOTUs was used to evaluate the richness of the gut viral community, and Shannon’s diversity index was used to estimate the diversity of the virome. Both the viral richness and diversity were calculated using the *vegan* package in the R platform.

### Functional annotation of the viral genomes

For functional annotation, we aligned the viral protein-coding genes for vOTUs against the Kyoto Encyclopedia of Genes and Genomes (KEGG) database (38) using DIAMOND (33) with the options “-query-cover 50 -subject-cover 50-e 1e-5 -min-score 50 -max-target-seqs 50.” Each protein was assigned a KEGG Orthology (KO) on the basis of the best-hit protein in the database. Viral auxiliary metabolic genes (AMGs) were identified according to the methods described in a previous study (39).

### Gut bacteriome profiling

We performed bacterial taxonomic profiling (including the phylum, class, order, family, genus, and species levels) of the fecal metagenomic data set for ACVD patients and healthy controls using MetaPhlAn 4 (database version: vJan21 CHOCOPhlAnSGB 202103.1) (40), which relies on clade-specific marker genes to unambiguously classify metagenomic reads to taxonomies and yield relative abundances of taxa identified in the sample. For each fecal sample, a uniform number of reads (10 million) were randomly selected to calculate the relative abundance of each bacterial species.

### Statistical analyses and visualization

Statistical analyses were implemented on the R v4.0.1 platform. The principal coordinates analysis (PCoA) of the Bray‒Curtis distance was performed and visualized using the *vegan* package. Permutational multivariate analysis of variance (PERMANOVA) was carried out with the *adonis* function of the *vegan* package after checking for differences in dispersion using the *betadisper* function (41), and the *adonis P* value was generated based on 1,000 permutations. The Wilcoxon rank-sum test was used to measure significant differences in diversity between the two cohorts, respectively. The Fisher exact test was used to evaluate significant differences in the occurrence of viral hosts and KOs. Before difference analysis, the features were filtered based on a minimum prevalence of 0.1 and further subjected to LOG transformation. MaAsLin2 (42) method was used for difference analysis, and confounding factors, such as individuals’ sex, age, and BMI, were removed. In addition, *q* values were used for multiple testing corrections and generated by the Benjamini‒Hochberg procedure. A *P* value (for a single test) or *q* value (for multiple testing) less than 0.05 was considered to indicate statistical significance. Random forest models were trained using the *randomForest* package (1,000 trees) to distinguish ACVD patients and healthy controls based on the abundance profiles of the differential viral signatures. Spearman’s correlation analysis was implemented to quantify the correlations between viruses and bacteria. Correlations with an absolute correlation coefficient >0.50 and Spearman’s correlation test *q* < 0.05 are shown in the correlation network. For each virus–bacterium pair, a correlation coefficient was calculated based on the relative abundances. The network was visualized using the *igraph* package in R.

## RESULTS

### Metagenomic-based delineation of the gut virome

To characterize the gut viral community in patients with ACVD, we analyzed the metagenomic sequencing data set from fecal samples of 214 patients and 171 healthy individuals (7). The human- and bacteria-derived metagenomic reads were removed, and the remaining reads for all samples were mapped into a virus catalog constructed from publicly available fecal metagenomes of Chinese populations (comprising 67,096 nonredundant vOTUs; see Materials and Methods) to generate the gut viral compositions. On average, 4.58% (±2.0%) of reads from the fecal metagenomes could be assigned as viral sequences, which covered a total of 10,153 vOTUs (with coverage >75% in these cohorts; see Materials and Methods) for subsequent analyses.

**FIG 1:**
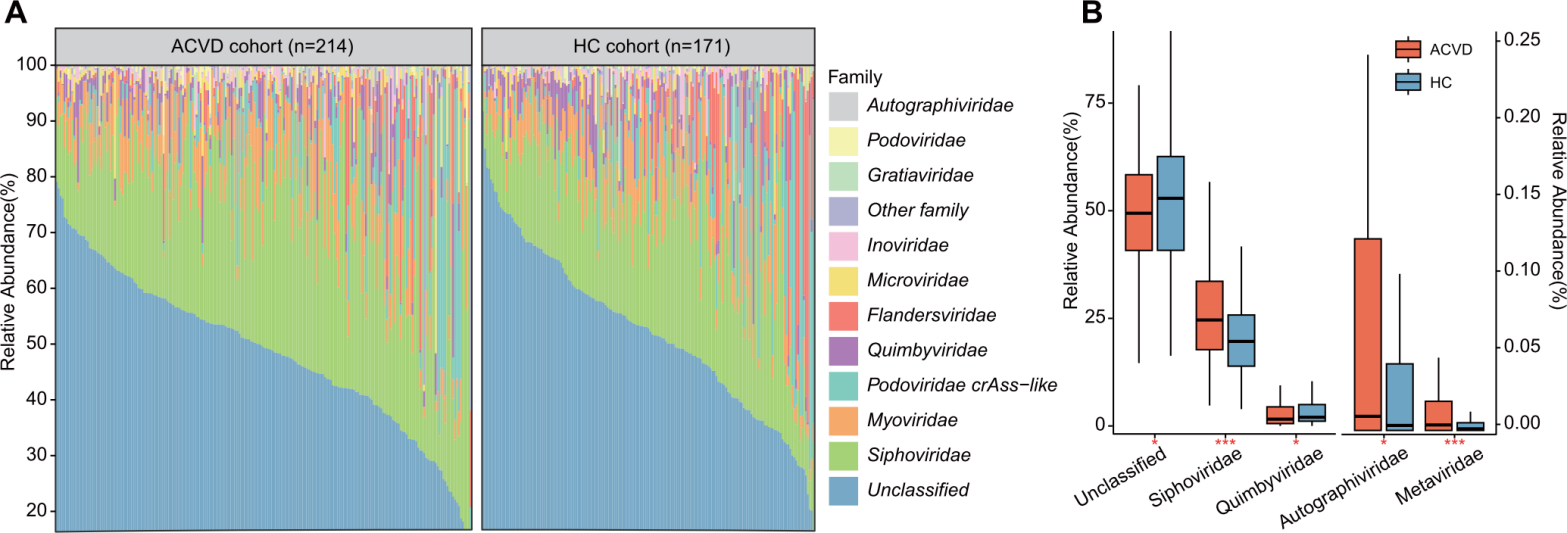
Family-level comparison of the gut virome between ACVD patients and healthy controls. (**A**) Bar plot showing the gut viral composition of fecal metagenomes from ACVD patients and healthy subjects at the family level. Only the top 10 viral families with the highest abundance are shown. (**B**) Boxplot showing the relative abundance of differentially abundant viral families between the two groups. Families enriched in the ACVD patients and healthy controls are labeled by orange and blue stars, respectively. The asterisks represent statistical significance (MaAsLin2 *q* value): * indicates *q* < 0.05, *** indicates *q* < 0.001.

At the family level, a large proportion of the total viral sequences were captured by vOTUs belonging to unknown viral families (Fig. 1A); this finding is consistent with previous studies (28, 43, 44) and highlights a considerable underrepresentation of the gut virome. We discovered that *Siphoviridae* (average relative abundance 26.7% vs 20.5% in patients vs healthy controls, MaAsLin2 *q* = 9.1 × 10^−6^) and *Myoviridae* were the most dominant families in all fecal samples, and the former was more abundant in the virome of ACVD patients (Fig. 1B; Table S2). The other low-abundance families included *Quimbyviridae*, *Podoviridae crAss-like*, *Microviridae*, *Flandersviridae*, *Podoviridae*, and several others. Compared with those of the healthy controls, *Metaviridae* and *Autographiviridae* showed a significant increase in abundance in the gut viral communities of the ACVD patients, whereas *Quimbyviridae* and Unclassified were significantly decreased in abundance (MaAsLin2 *q* < 0.05; Fig. 1B).

### Diversity and structural characteristics of the gut viral community in ACVD patients

**FIG 2:**
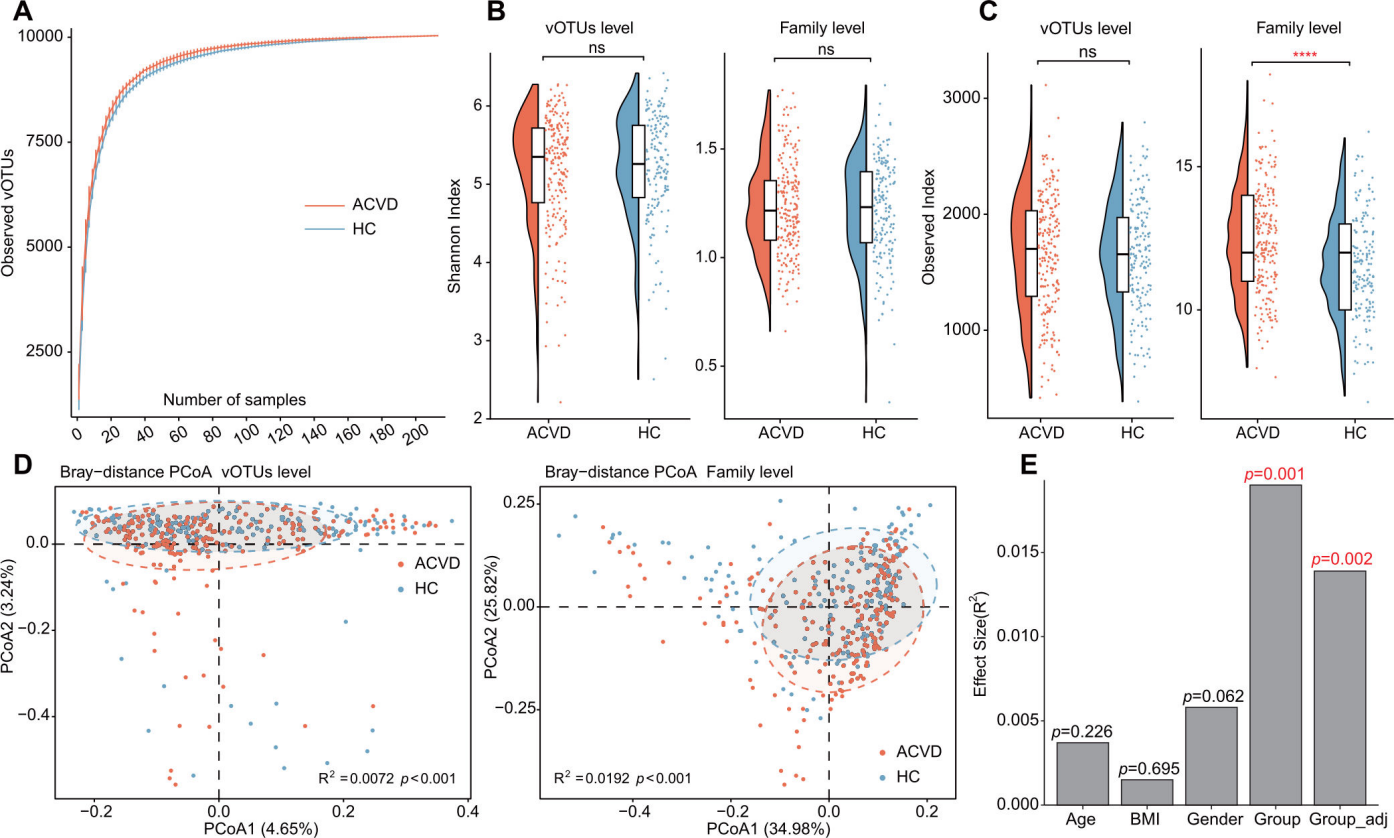
Diversity and multivariate analyses of the gut virome in ACVD patients and healthy controls. (A) Rarefaction analysis showed an increase in the number of vOTUs observed as the number of random samples increased. (B–C) Box and scatter plot showing the Shannon diversity index (B) and Richness index (C) of the gut virome of all samples. Both indexes are shown at the vOTU (left panels) and family (right panels) levels. Boxes represent the interquartile range between the first and third quartiles and the median (internal line). Whiskers denote the lowest and highest values within 1.5 times the range of the first and third quartiles, respectively; dots represent outlier samples beyond the whiskers. Wilcoxon rank-sum test: ns, not significant; ****, *P* < 0.0001. (D) PCoA of the Bray‒Curtis distance of the gut virome of all samples at the vOTU (left panels) and family (right panels) levels. Samples are shown at the first and second principal coordinates (PC1 and PC2), and the ratio of variance contributed by these two PCs is shown. Ellipsoids represent a 95% CI surrounding each group. (E) PERMANOVA results reveal the effect size of phenotype indexes and the ACVD state that contribute to the variance of the overall gut virome. Bar plots indicate the explained variation (effect size R^2^) of each phenotype factor. The effect size of the ACVD state after adjusting for sex, age, and BMI is also shown. *P* values were calculated using the *adonis* test with 1,000 permutations.

Rarefaction curve analysis of the gut virome revealed that the vOTU richness was not significantly different between the ACVD patients and healthy controls at the same sample sizes (Fig. 2A). Regarding the viral diversity of the vOTUs, we found that both the Shannon diversity index and Observed index were not significantly different between ACVD patients and healthy controls (Wilcoxon rank-sum test, *P* > 0.05; Fig. 2B through C). However, at the family level, ACVD patients exhibited a significantly higher level of viral family richness than healthy controls (Wilcoxon rank-sum test, *P* < 0.0001), whereas the Shannon index was not significantly different between groups.

We next used PCoA based on the Bray‒Curtis distance to further investigate the differences in the gut virome between patients and healthy controls. Clear separations were shown between the two groups at both the vOTU and family levels (Fig. 2D). PERMANOVA also revealed that the gut virome was significantly different between patients and healthy controls, with effect sizes of 1.90% (*adonis, P* < 0.001; difference in dispersion ANOVA, *P* > 0.05) at family level. Although PERMANOVA showed a significant difference between the two groups at the vOTU level (effect sizes of 0.72%, *P* < 0.001), the interpretation of the result was challenging due to the presence of a significant difference in sample dispersion between the two groups (ANOVA, *P* < 0.001) (41). Likewise, PERMANOVA of subjects’ phenotypes (i.e., sex, age, and BMI) showed that these attributes had almost no influence on the gut virome at the family level (effect size < 0.6% and *adonis, P* > 0.05;, Fig. 2E). After adjusting for all these phenotypes, the ACVD state was still significant, with an effect size of 1.4% (*adonis, P* < 0.002), supporting the conclusion that the ACVD state may independently impact the gut virome.

### Identification of ACVD-associated viral signatures

**FIG 3:**
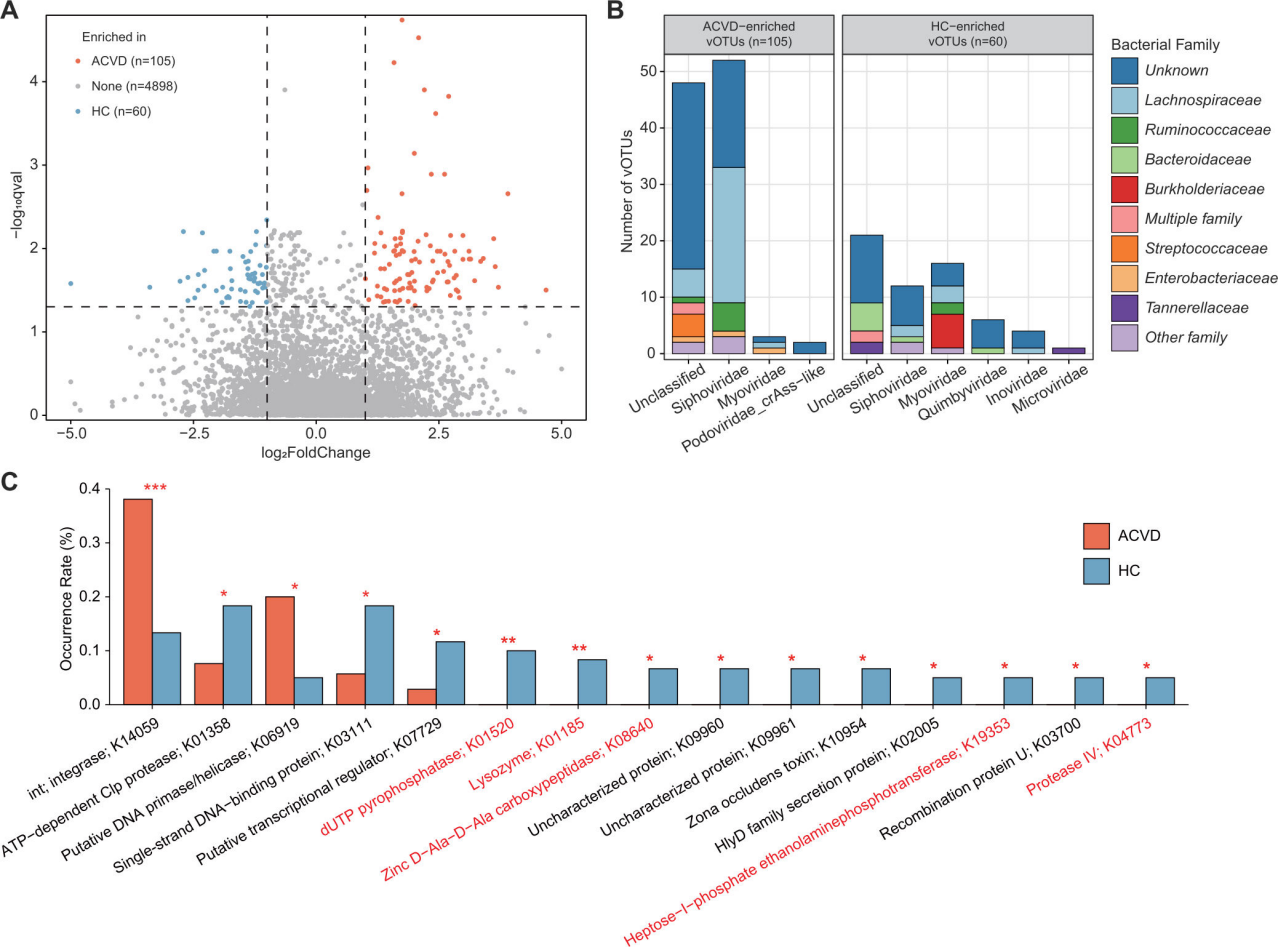
ACVD-associated gut viral signatures. (**A**) Volcano map showing the fold change and *Q* values of all vOTUs. vOTUs whose absolute value of fold change greater than 2 and *q* value less than 0.05 were considered significantly differentially abundant between ACVD patients and healthy controls, represented by orange and blue dots in the figure, respectively. (**B**) Bar accumulation plots show the taxonomical and predicted host distributions of vOTUs enriched in the ACVD and control groups. Viruses that are predicted to infect multiple bacterial families are labeled as “multiple families.” (**C**) Bar plot showing the occurrence rates of 15 differentially abundant KEGG orthologs (KOs) between the ACVD-enriched and control-enriched vOTUs. KOs that are viral auxiliary metabolic genes are indicated in red font. Fisher’s exact test: *, *P* < 0.05; **, *P* < 0.01; ***, *P* < 0.001.

To explore the gut viral signatures of ACVD, we compared the viral profiles between patients and healthy controls at the vOTU level. In total, 165 vOTUs were identified with significant differences in relative abundances between the two groups (MaAsLin2 *q* < 0.05 and |fold-change| > 2; Fig. 3A; Table S3). Among these, 105 vOTUs were enriched in the virome of ACVD patients, and 60 vOTUs were more abundant in healthy controls. The ACVD-enriched vOTUs included 52 members of *Siphoviridae* (corresponding to 49.5% of the 105 ACVD-enriched vOTUs), 3 *Myoviridae* (2.9%), 2 *crAss-like* (1.9%), 4 *Microviridae* (1.1%), and 50 family-unclassified viruses (47.6%) (Fig. 3B; Table S3). On the other hand, the control-enriched vOTUs were mainly composed of unclassified viruses (*n* = 23, corresponding to 38.8% of the 60 control-enriched vOTUs), followed by 16 *Myoviridae* (26.7%), 12 *Siphoviridae* (20.0%), and 6 *Quimbyviridae* (10.0%).

We next performed a host assignment of the vOTUs based on their homology or similarity of their CRISPR spacers to the prokaryotic genomes from the UHGG database (34). The analysis assigned 47.9% of the ACVD-associated vOTUs to one or more prokaryotic hosts. The ACVD-enriched vOTUs included a large number of predicted *Lachnospiraceae* (*n* = 30, corresponding to 28.6% of the 105 ACVD-enriched vOTUs), followed by *Streptococcaceae* (*n* = 6, 5.7%), *Ruminococcaceae* (*n* = 6, 5.7%), and *Enterobacteriaceae* (*n* = 3, 2.9%) phages (Fig. 3B; Table S3). Of these, no *Streptococcaceae*, *Lactobacillaceae*, and *Enterobacteriaceae* phages appeared in the control-enriched vOTUs. The control-enriched vOTUs contained 13.3% (11/67) *Bacteroidaceae*, 10% *Burkholderiaceae*, and 3.3% *Tannerellaceae* phages, whereas bacteriophages belonging to these families appeared seldomly in the ACVD-enriched vOTUs. In addition, *Bacteroidaceae* and *Burkholderiaceae* phages were significantly more prevalent in control-enriched vOTUs than in ACVD (Fisher’s exact test, *P* < 0.05).

We annotated the functions of the protein-coding genes of 165 ACVD-associated vOTUs using the KEGG database. A total of 15.8% (1,401/8,853) of genes from the vOTUs could be assigned to 452 KEGG orthologs (KOs). Of these, 15 KOs had significantly different occurrence rates between ACVD-enriched and control-enriched vOTUs, including 2 KOs that were more frequently present in the ACVD-enriched vOTUs and 13 KOs that were more likely to be encoded by control-enriched vOTUs (Fig. 3C; Table S4). Notably, we found that 4 of the 15 differentially abundant KOs were viral AMGs that are probably directly involved in host metabolism or other biological processes (45, 46). Almost all of these AMGs had a higher frequency in the control-enriched vOTUs than in the ACVD-enriched vOTUs (Fig. 3C; Table S4). This finding suggests that there is an obvious decrease in auxiliary metabolic biological functions in the gut virome when patients suffer from ACVD.

### Classification of ACVD status based on the gut virome

**FIG 4:**
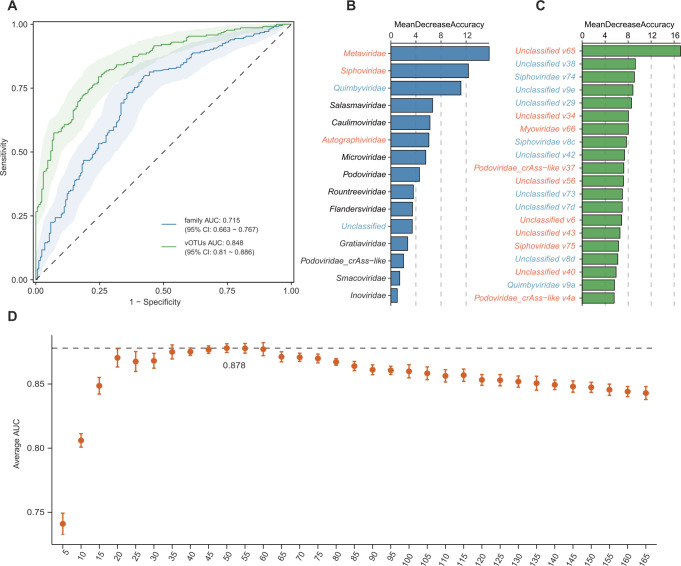
Gut virome-based classification of ACVD patients and healthy controls. (**A**) Random forest models for discriminating ACVD patients and healthy controls based on gut viral profiles at the vOTU and family levels. The area under the receiver-operating characteristic curve (AUC) and 95% CI are shown. (**B and C**) Mean decrease in the accuracy of the prediction based on viral families (**B**) and the 20 most important vOTUs (**C**) in the random forest models. Families and vOTUs that are enriched in the gut virome of patients and healthy controls are labeled orange and blue, respectively. (**D**) Exploring the classification performance for different numbers of viral signatures ordered in importance. Nodes show the average AUC of models with 10 repetitions under a specified number of vOTUs, and the error bars show the square deviations.

Next, we used the random forest classification model with 10-fold cross-validation to assess the performance of gut viral signatures in recognizing ACVD status. A model trained based on the relative abundances of the gut virome constituents at the family level achieved moderate classification ability in distinguishing ACVD patients from healthy controls, with an area under the receiver operator characteristic curve (AUC) of 0.715 (95% CI, 0.663–0.767; Fig. 4A through B). Moreover, the model trained by 165 ACVD-associated vOTUs reached a significantly higher discrimination power than the family-level model, with an AUC of 0.848 (95% CI, 0.810–0.886). The ACVD-enriched vOTU, *Unclassified v65*, featured the highest discrimination score in the random forest model, followed by *Unclassified v34, Myoviridae v66, Podoviridae crAss-like v37*, and others (Fig. 4C). Moreover, we trained new random forest models using the most important vOTUs to explore a minimal set of gut viral signatures for ACVD classification. This model using a subset of the top 50 most important vOTUs obtained the highest AUC of 0.878 (95% CI, 0.849–0.914) (Fig. 4D; Table S3). Collectively, these findings suggest that these gut viral signatures have the potential to differentiate ACVD patients from healthy controls.

### Correlations between gut viral signatures and bacteria

**FIG 5:**
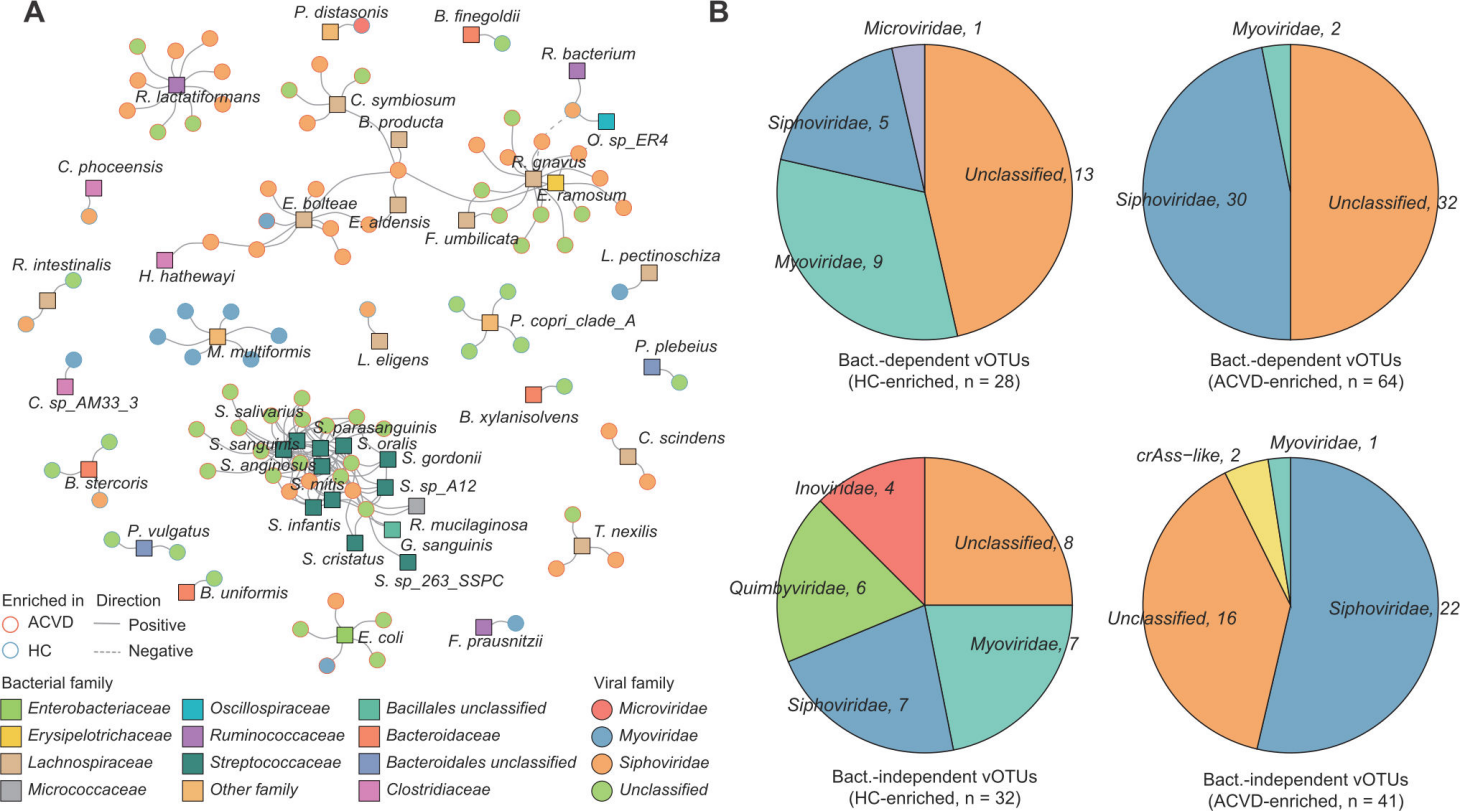
Correlation analysis between ACVD-associated vOTUs and gut bacteria. (**A**) Network showing the correlations of the ACVD-associated vOTUs and bacterial species. Spearman’s correlation coefficient was used to evaluate the correlation, and correlations with a correlation coefficient >0.50 and Spearman’s correlation test, *q* < 0.01 are shown in the network. (**B and C**) Pie plots show the taxonomic distribution of bacterium-dependent (upper panels) and bacterium-independent vOTUs (lower panels).

Finally, we wanted to investigate the connections between ACVD-associated gut viral signatures and the gut bacteriome. Using Spearman correlation coefficient analysis adjusted for individuals’ ACVD status, sex, age, and BMI, we identified a large number of co-abundance correlations between 92 ACVD-associated vOTUs (64 ACVD-enriched and 28 control-enriched vOTUs) and 42 bacterial species (coefficient > 0.50, *q* < 0.01; Fig. 5A). In general, nearly all of these vOTUs showed a positive correlation with gut bacteria, except for *Siphoviridae v99* and *v9d*, which exhibited a negative association with *Ruminococcus gnavus* and *Oscillibacter* sp. *ER4*, respectively. Several bacteria, such as *Streptococcus* spp. (*S. oralis*, *S. salivarius, S. anginosus*, *S. infantis*, *S. mitis*, *S. cristatus*, *S. gordonii*, *S. parasanguinis*, *S. sp. 263 SSPC*, and *S. sp. A12*), *Enterocloster_bolteae*, and *Ruminococcus gnavus*, were positively correlated with the highest number of ACVD-enriched vOTUs, whereas *Bacteroides* spp. (*B. finegoldii*, *B. stercoris*, *B. uniformis*, and *B. xylanisolvens*), *Mesosutterella_multiformis*, and *Prevotella copri* were correlated with control-enriched vOTUs.

In addition, unlike the 92 bacterium-dependent vOTUs (have a strong association with bacteria), the other viral signatures (including 41 ACVD-enriched and 32 control-enriched vOTUs) seemed to act on disease independently of bacteria (Fig. 5B through C). Taxonomically, the bacterium-independent vOTUs (which do not have strong association with bacteria) were more frequently present in the *Quimbyviridae, crAss-like*, and *Inoviridae* viruses than the bacterium-dependent vOTUs; their roles in disease also need further study.

## DISCUSSION

In this study, we performed a deep metagenomic-based analysis of the virome of fecal samples from a population of 214 ACVD patients and 171 healthy controls. To our knowledge, this is the first study investigating the gut virome in patients with ACVD. Consistent with previous observations from the gut bacteriome (7), the gut virome of ACVD patients was distinctly different from that of healthy controls. At the family level, several of the most prevalent viral families are in the human gut. Among these, *Siphoviridae* was significantly enriched in the virome of ACVD patients. *Siphoviridae* belong to the dsDNA virus order *Caudovirales*, and in general, most members of this family are temperate viruses in the gut ecosystem (19, 47). The high relative abundance of these families suggests a potential high viral load in ACVD patients. However, the most representative control-enriched family was *Quimbyviridae*, which is a recently described viral family known for its high abundance, prevalence, and hypervariability in the human gut, and this family is predicted to infect *Bacteroidetes* (32). Considering that *Quimbyviridae* have large genomes that encode a wide range of functions, the lower relative abundance of these phages may lead to the absence of certain important functions in the gut virome of patients with ACVD. We identified 105 vOTUs that were enriched in ACVD patients and 60 vOTUs that were enriched in healthy controls. Host prediction of these viral signatures indicated that a large proportion of ACVD-enriched vOTUs were predicted to infect *Enterobacteriaceae*, *Lachnospiraceae*, and *Streptococcaceae*. Similarly, correlation analysis showed that gut *Escherichia coli*, *Enterocloster bolteae*, *Streptococcus* spp., and *Ruminococcus gnavus* were widely correlated with ACVD-enriched vOTUs. Increased abundance of Enterobacteriaceae phages was also observed in the gut virome of patients with ulcerative colitis (48) or alcoholic hepatitis (49). In patients with liver disease, the increased abundance of lysogenic Enterobacteriaceae phages could facilitate the growth of pathogenic bacterial species (50). As observed in a gut bacteriome study, the abundances of Enterobacteriaceae and *Streptococcus* in ACVD patients were also increased (7). Therefore, the coincidently high levels of these viruses and bacteria might reflect that the viruses depend on the gut bacteria to impact disease status. Conversely, many control-enriched vOTUs were predicted to be phages of Bacteroidaceae and were frequently associated with the gut bacterium *P. copri*. Bacteroidetes members in the human gut are responsible for breaking down most plants (mainly by *Prevotella* spp.) and animal polysaccharides (mainly by *Bacteroides* spp.) and have the capacity to produce beneficial bioactive molecules such as B-complex vitamins (51, 52). Correspondingly, the phages of these bacteria may also play roles in these processes. In other words, it is possible that some of the viral sequences identified in the gut virome analysis could be integrated into bacterial genomes as proviruses. This would mean that the observed differences in virome composition between disease and control groups might actually reflect differences in the bacterial hosts harboring these proviruses. In this scenario, the viral presence, absence, or abundance may affect disease progression by influencing the behavior or regulation of the bacterial host. In addition, we found that a proportion of ACVD-associated viruses (41 ACVD-enriched and 32 control-enriched vOTUs) did not have a strong correlation with gut bacteria; they may act in the disease independently of the gut bacteriome.

Functional analysis of the ACVD-associated vOTUs revealed that many viral AMGs were enriched in control-enriched vOTUs, suggesting that the ability of the viruses to assist the host mechanism was decreased in patients with ACVD. Specifically, the identified AMGs are involved in amino acid, nucleotide, and other metabolic processes. This finding raises intriguing possibilities regarding the role of these viral AMGs in host metabolism and disease progression. It suggests that the altered abundance or functionality of these viral AMGs may contribute to metabolic dysregulation or imbalances observed in ACVD patients. However, further investigations would be required to elucidate the precise mechanisms underlying this association and explore potential therapeutic implications. We trained random forest models based on the gut viral signatures for disease discrimination and achieved an optimal AUC of 0.878 for distinguishing patients from healthy controls. This discriminatory power was comparable with that from the prediction model based on bacterial signatures (AUC = 0.86) (7). Our results thus highlighted the diagnostic potential of the gut virome in ACVD and related diseases. In a separate study conducted by Tisza et al. as mentioned (53), the characteristics of the gut virome in ACVD patients were investigated using the Cenote Human Virome Database they constructed, which encompassed approximately 45,000 unique virus taxa. Compared to the findings of Tisza et al., our study identified a higher number of viral markers that differentiated between ACVD patients and healthy controls. This suggests that our analysis was more sensitive in detecting viral signatures associated with ACVD, based on the gut virus catalog we constructed. However, due to the limitations of the relevant data sets, a more in-depth comparison could not be performed. It is worth noting that the higher discriminative ability observed in our study may be attributed to the fact that both the metagenome samples and reference database used in our analysis were sourced from China. The gut virome composition can vary across different geographical regions due to factors such as diet, lifestyle, and environmental factors (54). By utilizing data from the Chinese gut virome catalog, we were able to capture a more specific and representative virome profile related to ACVD in the Chinese populations. Due to the lack of comprehensive reference databases, the majority of viruses in the human gut microbiota remain unknown; this is a major limitation of our gut virome study. Many unclassified viruses are taxonomically classified into known or newly identified taxa, which results in more accurate descriptions of the virome characterization of ACVD patients. On the other hand, unlike whole-metagenome-based technology, virus-like particle (VLP) virome technology has shown the generation of more abundant viral sequences and illumination of the characteristics of the virome in multiple diseases (48, 55, 56), which may also facilitate future studies of the ACVD virome.

### Conclusion

In summary, we characterized the community diversity and structure of the gut virome of ACVD patients by comparison with that of healthy controls; identified numerous differentially abundant viral families, species, and functions in relation to ACVD; and identified potential gut viral dysbiosis in patients. Identification of these gut viral signatures may facilitate further mechanistic, diagnostic, and interventional studies of ACVD and related diseases.

## Data Availability

The original contributions presented in the study are included in the article/supplemental material; further inquiries can be directed to the corresponding author.

## SUPPLEMENTAL MATERIAL

Table S1 spectrum.01050-23-s0001.xlsx Metadata of the ACVD data sets and read mapping rate for multiple references.

Table S2 spectrum.01050-23-s0002.xlsx Comparison of the gut virome between ACVD patients and healthy controls at the family level.

Table S3 spectrum.01050-23-s0003.xlsx Detailed information of 419 ACVD-associated vOTUs.

Table S4 spectrum.01050-23-s0004.xlsx Detailed information of 22 KOs that significantly differed in occurrence frequency between ACVD-enriched and control-enriched vOTUs.

ASM does not own the copyrights to Supplemental Material that may be linked to, or accessed through, an article. The authors have granted ASM a non-exclusive, world-wide license to publish the Supplemental Material files. Please contact the corresponding author directly for reuse.
